# Morphine Withdrawal Modifies Prion Protein Expression in Rat Hippocampus

**DOI:** 10.1371/journal.pone.0169571

**Published:** 2017-01-12

**Authors:** Vincenzo Mattei, Stefano Martellucci, Francesca Santilli, Valeria Manganelli, Tina Garofalo, Niccolò Candelise, Alessandra Caruso, Maurizio Sorice, Sergio Scaccianoce, Roberta Misasi

**Affiliations:** 1 Laboratorio di Medicina Sperimentale e Patologia Ambientale, Polo Universitario di Rieti “Sabina Universitas”, Rieti, Italia; 2 Dipartimento di Medicina Sperimentale, Università di Roma “La Sapienza”, Roma, Italia; 3 Dipartimento di Fisiologia e Farmacologia "Vittorio Erspamer”, Università di Roma “La Sapienza”, Roma, Italia; University of Melbourne, AUSTRALIA

## Abstract

The hippocampus is a vulnerable brain structure susceptible to damage during aging and chronic stress. Repeated exposure to opioids may alter the brain so that it functions normally when the drugs are present, thus, a prolonged withdrawal might lead to homeostatic changes headed for the restoration of the physiological state. Abuse of morphine may lead to Reacting Oxygen Species-induced neurodegeneration and apoptosis. It has been proposed that during morphine withdrawal, stress responses might be responsible, at least in part, for long-term changes of hippocampal plasticity. Since prion protein is involved in both, Reacting Oxygen Species mediated stress responses and synaptic plasticity, in this work we investigate the effect of opiate withdrawal in rats after morphine treatment. We hypothesize that stressful stimuli induced by opiate withdrawal, and the subsequent long-term homeostatic changes in hippocampal plasticity, might modulate the Prion protein expression. Our results indicate that abstinence from the opiate induced a time-dependent and region-specific modification in Prion protein content, indeed during morphine withdrawal a selective unbalance of hippocampal Prion Protein is observable. Moreover, Prion protein overexpression in hippocampal tissue seems to generate a dimeric structure of Prion protein and α-cleavage at the hydrophobic domain. Stress factors or toxic insults can induce cytosolic dimerization of Prion Protein through the hydrophobic domain, which in turn, it stimulates the α-cleavage and the production of neuroprotective Prion protein fragments. We speculate that this might be the mechanism by which stressful stimuli induced by opiate withdrawal and the subsequent long-term homeostatic changes in hippocampal plasticity, modulate the expression and the dynamics of Prion protein.

## Introduction

Prion protein (PrP^C^) is a glycosylphosphatidylinositol (GPI) anchored protein found in the outer leaflet of the plasma membrane [1]. It is present in body fluids and in the plasma membrane of neural and lymphocytic cells [1,2,3].

The cellular form of PrP^C^ is a highly conserved cell surface GPI-anchored glycoprotein that was first identified as molecule able to bind Cu^2++^ *in vitro* [4].

Like many GPI-anchored protein, PrP^C^ is found in sphingolipid-rich membrane microdomains known as lipid raft [3,5] and, beyond them, into post-synaptic densities [6]. This membrane-bound isoform dimerizes and can act as cell surface receptor, as a co-receptor, or form multimolecular complexes and thus recruit downstream signal transduction pathways [7].

PrP^C^ has been extensively investigated because its Scrapie isoform, PrP^Sc^, leads to the development of Transmissible Spongiform Encephalopaty (TSE), a class of fatal neurodegenerative disorders that affects several mammals [8]. In brief, according to the protein-only hypothesis [1], the Scrapie isoform, acting as a seed, promotes the conversion of the unstructured N-terminal domain of the PrP^C^ into a β-sheet rich structure (with the help of a supposed protein X). This misfolded domain binds to- and aggregates with- others PrP^C^, promoting their conversion and establishing a positive feedback until PrP^C^ is converted into the pathogenic PrP^Sc^ isoform. The exact mechanism of this conformational change or prion conversion is unclear but may involve the initial formation of dimers.

Several observations indicate that PrP can form dimers, both in its physiologic and pathologic state [9,10,11].

PrP^C^ was shown to be involved in a plethora of different physiological functions, including cytoskeleton and neurites regulation [12], memory consolidation [13,14,15], synaptic functions [16,17,18] and neuroprotection, the latter of which is the most thoroughly studied function of PrP^C^. During the late stage of the secretory pathway, PrP^C^ can undergo a cleavage at position 111/112 in its hydrophobic domain, termed α-cleavage, which produces a soluble amino-terminal fragment, termed N1, and a membrane-bound C-terminal fragment, termed C1 [19,20,21,22]. Several recent findings highlighted the physiological importance of this event, since both these metabolites may exert a neuroprotective function. Moreover, PrP-N1 and PrP-C1 production is stimulated as a consequence of intracellular dimerization. According to this model, stress factors or toxic insults might induce prion cytosolic dimerization through the hydrophobic domain. In turn, the dimerization stimulates α-cleavage and thus the production of the neuroprotective fragments [23].

Overall, PrP^C^ appears to be related with almost every aspects of neuronal physiology. Dimerization of membrane-bound PrP^C^ leads to clustering in multimolecular complexes and serves to regulate different aspect of neuronal homeostasis, while intracellular dimerization appears to be the most relevant event in neuroprotection, via N1 and C1 prion metabolites.

Moreover, it has been shown that PrP^C^ has a similar activity than superoxide dismutase (SOD) [24,25], and may act as a free radical scavenger [26], thereby contributing to the antioxidative capacity of cells. There is increasing evidence that PrP^C^ plays a role in the cellular resistance to oxidative stress, being involved in/or dependent on copper metabolism in brain [4]. Infact, PrP^C^ null mice show reduced resistance to oxidative stress, presumably owing to either decreased of Cu/Zn SOD [27] and/or decrease in glutathione reductase activity, which is involved in the generation of reduced glutathione (GSH) [28]. An additional cleavage of PrP^C^ may occur upstream α cleavage, the β cleavage, which produces soluble N2 and membrane-bound C2 fragments [29]. β cleavage has been proposed to have a fundamental role in the mechanism by which PrP^C^ protects cells against oxidative stress [30].

Several scientists investigated the effect of morphine abuse on the central nervous system (CNS) neurodegeneration [31].

Plastic changes during opiate withdrawal have been associated also with stress responses [32]. In particular, it has been proposed [33] that during morphine withdrawal, stress responses might be responsible, at least in part, for long-term changes of hippocampal plasticity and affect metaplasticity [34], defined as the phenomenon that influences the direction and the threshold for the subsequent induction of synaptic plasticity [35].

Since PrP^C^ is involved in both ROS mediated stress responses [36,37] and in synaptic plasticity [38]. We speculate that stressful stimuli induced by opiate withdrawal, and the subsequent long-term homeostatic changes in hippocampal plasticity, might modulate the expression of PrP^C^. In this work we investigate the effect of withdrawal in rats after a chronic morphine treatment, we evaluated the generation of PrP^C^ oligomeric species, such as dimers, which could further aggregate into resistant form of PrP.

## Materials and Methods

### Experimental design and drug treatment

#### Animals

Male Sprague–Dawley rats (Harlan, Italy) weighing 200–220 g at the beginning of treatment. They were kept in a temperature-controlled room (24 ± 2°C), on a 12-h light and 12-h dark period (lights on at 7am). Food (Standard Diet 4RF21, Charles River, Massachusetts, USA) and tap water were provided ad libitum. The animals were housed in standard methacrylate cages (two rats per cage) with flat floor covered with sawdust, which was changed weekly. Experimental protocols were performed in strict accordance with the European (86/609/EEC) and Italian (DLgs 116/92) guidelines on animal care. Animals were killed by decapitation. All efforts were made to minimize animal suffering during the experiments. Our protocol was submitted to the Ethics Committee of Italian Ministry of Health, which specifically approved the protocol of this study on December 29, 2006, Authorization n° 181/2006-B to S.S.

#### Treatments

**Fig 1:**
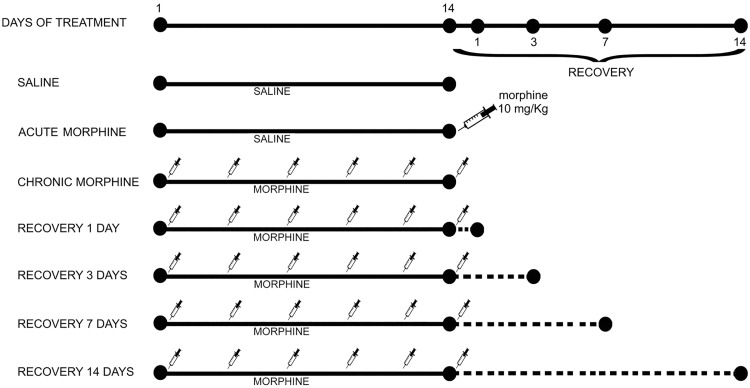
Scheme of morphine treatment in rats. Seven groups of male rats were used in the study. Acute morphine group was treated with saline twice daily for 14 days and subject to morphine administration 1 h before killing. Chronic morphine group morphine was administered twice daily for 14 days, last morphine administration was 1 h before killing. For all recovery groups (1, 3, 7 and 14 days) morphine was administered twice daily for 14 days, last morphine administration was 1, 3, 7, 14 days before killing. In saline group, saline instead of morphine was used.

Morphine hydrochloride (SALARS, Como, Italy) was dissolved in saline and administered twice daily (at 8am and 8pm) for 14 days, as previously described [39,40]. Briefly, the initial dose administered was 10 mg/kg and it was increased by 20 mg/kg every other day until the 14th day of treatment. Control rats received an equal volume of saline. Rats were treated for 14 days intraperitoneally and then assigned to one of the following groups (n = 6) (Fig 1):

1. chronically treated with saline, twice daily for 14 days;
2. chronically treated with saline, twice daily for 14 days, morphine admistration (10 mg/Kg) 1 h before killing (AM);
3. chronically treated with morphine twice daily for 14 days, last morphine administration was 1 h before killing (CM);
4. chronically treated with morphine twice daily for 14 days, last morphine administration was 1 day before killing (CM + 1);
5. chronically treated with morphine twice daily for 14 days, last morphine administration was 3 days before killing (CM + 3);
6. chronically treated with morphine twice daily for 14 days, last morphine administration was 7 days before killing (CM + 7).
7. chronically treated with morphine twice daily for 14 days, last morphine administration was 14 days before killing (CM + 14).

No animal death was observed during treatment.

### Antibodies

Mouse anti-PrP SAF32 monoclonal antibody (Spi-Bio, Bertin Pharma, France) and mouse anti-PrP SAF61 monoclonal antibody (Spi-Bio, Bertin Pharma, France) which recognized prion protein at different epitopes (SAF32 a.a. 79–92; SAF 61 a.a.144–160), goat anti-PrP C-20 polyclonal antibody or mouse anti-Thy-1 monoclonal antibody (Abcam, Cambridge, UK) were employed. Bound antibodies were visualized with horseradish peroxidase-conjugated anti-mouse IgG (Amersham Biosciences, Uppsala, Sweden) or anti-goat IgG (Sigma-Aldrich, Milan, Italy)

### Brain dissection

Rats were killed at the end of the treatments as indicated in Fig 1 by decapitation, the hippocampus and prefrontal cortex were dissected and frozen. Tissues were weighed and homogenized in lysing solution containing 1% Nonidet, 0.1 M Tris pH 8.0, 0.15M NaCl, 5 nM ethylenediaminetetraacetic acid (EDTA), 1mM phenyl metyl sulfonyl fluoride (PMSF), 1mM Sodium orthovanadate (Na_3_VO_4_). Homogenized tissues were subjected to sodium dodecyl sulfate-10% polyacrylamide gel electrophoresis (SDS-PAGE) and western blot.

### SDS-PAGE and western blot analysis

Homogenized hippocampi and prefrontal cortices (ca.30 μg of total proteins) were subjected to sodium-dodecyl sulphate 10% or 15% SDS-PAGE. Two different western blots were probed with anti-PrP SAF32 (Spi-Bio, Bertin Pharma, France) or anti-PrP SAF61 (Spi-Bio, Bertin Pharma, France) which recognized PrP^C^. Bound antibodies were visualized with horseradish peroxidase-conjugated anti-mouse IgG (Amersham Biosciences, Uppsala, Sweden) and immunoreactivity assessed by chemiluminescence reaction using the ECL Western blotting detection system (Amersham, UK). PrP^C^ bands were subjected to densitometric scanning analysis, performed by Mac OS X (Apple Computer International), using NIH Image 1.62 software. ImageJ densitometry software (Version 1.6, National Institutes of Health, Bethesda, MD) was used for band quantitative densitometric analysis. Selected bands were quantified on the basis of their relative intensities, which are reported as arbitrary densitometric units. The software allows the measurement of density profiles, peak heights as well as peak intensity (average OD of the band, INT) or volume (average OD of the band times its area, INT*mm^2^) of the band.

### Protease resistance assay with proteinase K

Homogenized hippocampi (ca. 30 μg of total proteins) were incubated with proteinase K (5 μg/ml) (Sigma-Aldrich, Milan, Italy) for 30 minutes at 37°C and subjected to 10% SDS-PAGE). Western blots were probed with anti-PrP SAF32 monoclonal antibody (Spi-Bio, Bertin Pharma, France).

### Protein deglycosylation

Thirty μg of protein were denaturated and incubated with 0,5 U/ml of peptide N-glycosidase (PNGase) (Sigma-Aldrich, Milan, Italy), at 37°C for 2h. The reaction was stopped by adding an equal volume of sample buffer 2X (Sigma-Aldrich, Milan, Italy). Samples were then resolved on a 15% SDS-PAGE gel for Western Blot analysis. The blot was probed with anti-PrP SAF61 monoclonal antibody and, as a control, with anti-PrP SAF32 monoclonal antibody.

### Statistical analysis

Quantitative analysis of immunoblot images was carried out using NIH Image 1.62 as software (Mac OS X, Apple Computer International). Data were analyzed using one-way analysis of variance (ANOVA) after Bartlett’s test for the homogeneity of variances and Kolmogorov-Smirnov’s test for the Gaussian distribution and followed by Newman-Keuls multiple-comparison test or, when appropriate, with Student’s *t*-test. All data reported were verified in at least three different experiments and reported as mean ± SD. Only p values <0.05 were considered as statistically significant.

## Results

### Effects of acute and chronic morphine treatment on PrP^C^ expression

**Fig 2:**
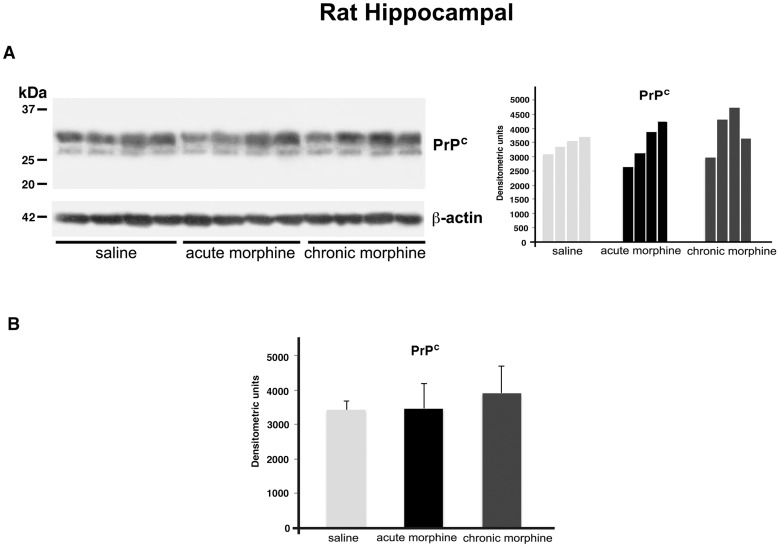
PrP^C^ expression in the hippocampus of rats treated with morphine. Representative immunoblot of PrP^C^ and β-actin from hippocampus (A) of saline-treated rats and rats treated with morphine (see Materials and Methods for details). Densitometric analysis of bands from the representative western blot is shown in panel on the right. Densitometric analysis of bands from each group of samples is reported in panel B, as Mean ± SD. Morphine treatment was NSS vs Saline.

In a first series of experiments, we investigated the effects of both acute and chronic morphine exposure (see material and method) on PrP^C^ expression in rats hippocampus and prefrontal cortex (not shown). The results (Fig 2) showed a double band of about 30kDa, recognized by anti-PrP SAF32 monoclonal antibody. However, densitometric analysis (Fig 2B) demonstrated that opiate administration did not affect PrP^C^ expression either in hippocampus or prefrontal cortex.

### Effects of morphine withdrawal on PrP^C^ expression

**Fig 3:**
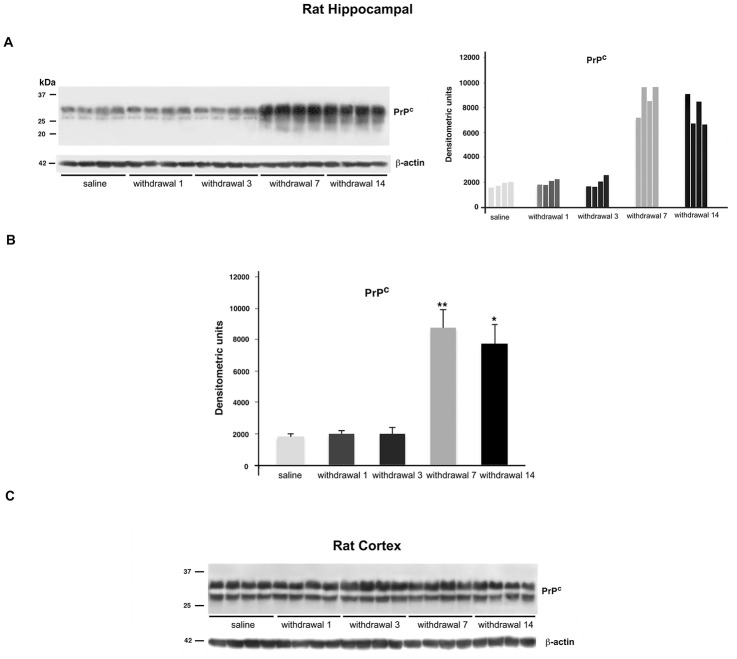
PrP^C^ content in the hippocampus and prefrontal cortex during morphine withdrawal. A: Representative immunoblot of PrP^C^ and β-actin of hippocampus from rats treated with morphine and subsequent withdrawal (left panel) and densitometric analysis (right panel). B: Densitometric analysis of bands from each group of samples, Mean ± SD, *P<0.05; **P<0.01 versus the corresponding saline. C: Immunoblot of prefrontal cortex from rats chronically treated with morphine and subsequent opiate withdrawal (see Materials and Methods for details).

Next, we analyzed whether opiate abstinence could influence PrP^C^ expression. Rats subjected to chronic morphine treatment and subsequent withdrawal of drug, showed a time-dependent increase of PrP^C^ hippocampal content. In fact, although 1 or 3 days of withdrawal did not influence protein expression, 7 and 14 days of withdrawal induced a marked (ca. 3 fold) increase of PrP^C^ content (Fig 3A and 3B). On the contrary, PrP^C^ expression in the prefrontal cortex was unaffected by morphine withdrawal (Fig 3C).

The effect of morphine withdrawal on PrP^C^ appeared to be quite specific, since the expression of the control GPI-anchored protein Thy-1 was virtually unaffected (S1 Fig).

### Dimeric form of PrP^C^ during morphine withdrawal

**Fig 4:**
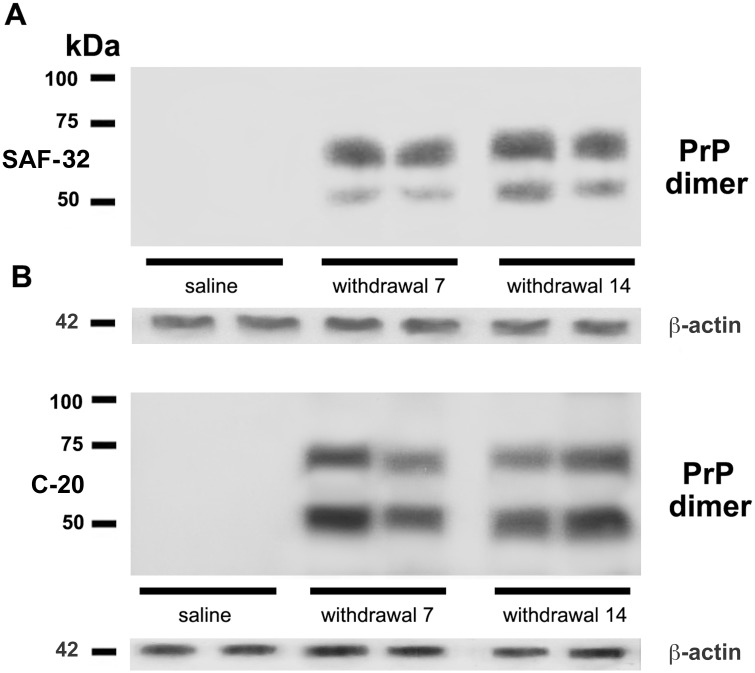
Evidence of PrP^C^ dimers in the hippocampus after morphine withdrawal. Representative immunoblots of PrP^C^ dimers from hippocampi of rats after 7 and 14 days of opiate withdrawal. Samples were analyzed by Western blot, using either anti-PrP SAF32 (A), or C-20 (B).

Since PrP^C^ dimerization plays a prominent role in PrP^C^ physiology, we investigated the presence in the hippocampus of this dimeric structure in PrP^C^ overexpressing tissues, resulting from morphine withdrawal (i.e. 7 and 14 days). With this purpose, we run a 10% SDS-PAGE in order to better analyze proteins in a range of 40–90 kDa. Indeed, it allowed us to separate two bands, the larger of approximately 60 KDa, and the smaller of about 48 kDa, in 7 and 14 days withdrawal samples. These bands in Western blot analysis specifically reacted with anti-PrP SAF32 (Fig 4A) and anti-PrP C-20 (Fig 4B).

### Proteinase K sensitivity of PrP^C^

**Fig 5:**
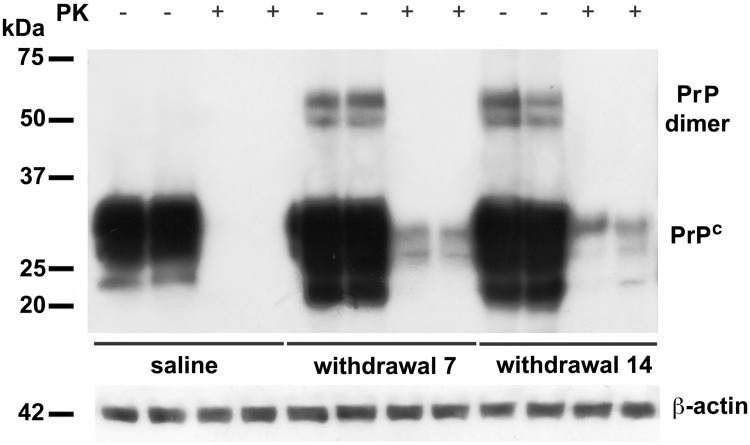
Proteinase K sensitivity of PrP^C^. Hippocampal samples derived from control (saline) and morphine-withdrawal rats (7 and 14 days after last morphine injection) were subjected to digestion with proteinase K (PK) 5 mg/ml. Note the bands at 30 and 26 kDa of undegraded PrP^C^, in comparison to control samples.

It has been suggested that oligomeric forms of PrP^C^, such as dimers, could facilitate a more rapid conversion of PrP^C^ Proteinase K (PK) sensitive into PrP Proteinase K resistant (PrP^res^) [41]. Thus, PK sensitivity was assessed in hippocampal samples overexpressing PrP^C^ (i.e. 7 and 14 days of morphine withdrawal). Homogenized samples were exposed to high concentration of PK (5 μg/ml) and then assayed by immunoblot, using the monoclonal antibody SAF32. The results showed that in both groups the bands at 48 and 60 kDa were completely digested (Fig 5). A little portion of undegraded PrP^C^ after proteinase K treatment was also detected at withdrawal 7 and 14, although PrP^C^ dimerization appears to be proteinase K sensitive (Fig 5).

### On the α-cleavage of PrP^C^ in chronic morphine treatment and withdrawal of drug

**Fig 6:**
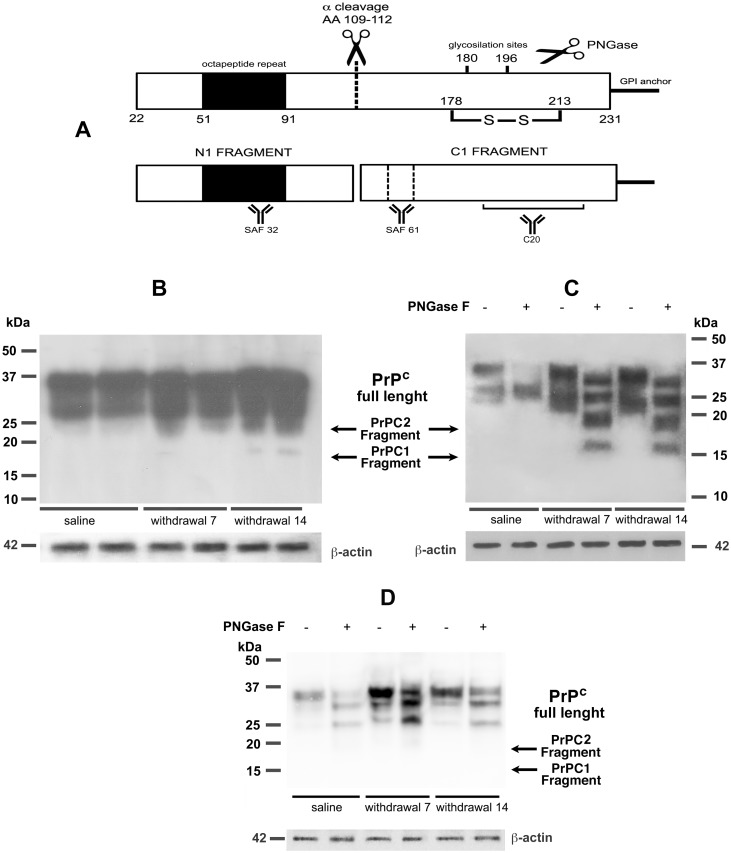
α-cleavage of PrP^C^. A: Scheme of α cleavage sites of the prion protein and its products, N1 and C1 fragments. Antibody binding sites of N1 and C1 fragments are showed; B: Hippocampal samples derived from control (saline) and morphine-withdrawal rats (7 and 14 days after last morphine injection) were analyzed using an anti-PrP Antibody SAF61. Note the presence of one band at approximately 18 kDa; C: Hippocampal samples derived from control (saline) and morphine-withdrawal rats (7 and 14 days after last morphine injection) were analyzed using an anti-PrP antibody SAF61 in the presence or absence of PNGase (0.5 U/ml). Note the bands at 30, 25, 18 and 16 kDa; D: Hippocampal samples derived from control (saline) and morphine-withdrawal rats (7 and 14 days after last morphine injection) were analyzed using an anti-PrP antibody SAF32 in the presence or absence of PNGase (0.5 U/ml). Note the bands at 37, 35 and 27 kDa.

It has been demonstrated that PrP^C^ homodimerization is an important regulator of PrP^C^ α-cleavage [23]. Proteolytic process of PrP^C^ under physiological condition includes α-cleavages resulting in a membrane-bound C-terminal (C1) fragment and a soluble N-terminal (N1) fragment (Fig 6A). To verify the possibility of α-cleavage of PrP^C^ in ours hippocampal samples, we performed a western blot with SAF61 antibody, which is able to recognize C1 fragment. In particular, the reactivity of the monoclonal antibody SAF61 highlighted (among the typical PrP^C^ pattern) the presence of one band at approximately 18 kDa, only in the 7 and 14 days withdrawal samples compared to saline (Fig 6B). In order to understand whether these bands could be produced by an α-cleavage, we repeated the same experiment in the presence or absence of PNGase (0,5 U/ml). The results showed that control samples show a typical band shift upon PNGase treatment, while in 7 and 14 days of withdrawal samples several bands were evident at 30, 25, 18 and 16 kDa (Fig 6C). The 16 kDa band is coherent with the molecular weight for the C1 fragment. Either way, this result suggests that, during morphine withdrawal, PrP^C^ undergoes post-translational endoproteolytic events that may lead to the production of the neuroprotective metabolites N1 and C1. Moreover, the highest molecular weight band in Fig 6C following PNGaseF treatment may represent an incomplete PNGaseF digestion of glycosylation, which could be consistent with PrP^C^ overexpression under our experimental condition. In addition, in Fig 6C a 18 kDa band compatible with the β cleavage process is detectable. We cannot exclude that in our samples a β cleavage occurs too, indeed, PrP^C^ is cleaved at the end of the copper-binding octapeptide repeats through the action of Reactive Oxygen Species (ROS), β cleavage, and their products, C2/N2 fragments, have been reported to be involved in stress protection as well [30].

In order to assure the identity of the 16 and 18 kDa bands, we performed a western blot of the same PNGase treated and untreated samples with Saf32 Ab, which recognizes the N terminal portion of the molecule at the octapeptide repeat (Fig 6D). This blot allowed us to identify in withdrawal samples upon PNGase treatment, three bans having MW between 27 and 37 kDa, which exclude the presence of N3 fragment (supposed to have approximately 20 kDa MW), as product of PrP^C^ γ cleavage.

## Discussion

The main findings of the present study can be summarized as follow: a) although neither acute, nor chronic morphine exposure, influenced PrP^C^ expression in hippocampus or prefrontal cortex, abstinence from the opiate induced a time-dependent and region-specific modification in PrP^C^ content. In fact, one week after morphine withdrawal, PrP^C^ expression in the hippocampus, but not in the prefrontal cortex, was significantly increased, and this effect was still present 14 days after last morphine exposure; b) this PrP^C^ overexpression in hippocampal tissue may be linked to the generation of the dimeric structure of PrP^C^ and c) opiate withdrawal leads to α-cleavage at the PrP^C^ hydrophobic domain and consequent PrPN1/C1 fragments production.

These data seem to go in the same vein of previous studies. Indeed, as already reported, PrP^C^ homodimerization through the hydrophobic domain stimulates the α-cleavage and thus the production of neuroprotective PrP^C^ fragments [23]. We speculate that this might be the mechanism by which stressful stimuli induced by opiate withdrawal and the subsequent long-term homeostatic changes in hippocampal plasticity, modulate the expression and the dynamics of PrP^C^. At present, the mechanism of the increase in PrP^C^ expression is not addressed at all. However, it was shown that, following different stress stimuli, no change in PrP mRNA levels was found. This suggests that treatment with stress stimuli resulted in a decrease in protein turnover, presumably via the proteasome pathway [42]. An inhibition of the proteasome in response to oxidative stress has also been shown [43].

Recently, the role of PrP^C^ has been investigated under oxidative stress and endoplasmic reticulum stress in neural damage. Results have indicated that PrP^C^ exerts a proapoptotic role during endoplasmic reticulum stress, but an anti-apoptotic role during oxidative stress-induced cell death. It suggests that PrP^C^ enhances the susceptibility of neural cells to impairment of protein processing and trafficking, but decreases the vulnerability to oxidative insults [44]. Moreover, brain contains low levels of antioxidant enzymes and high levels of easily oxidized substrates, which make the brain highly susceptible to oxidative damage [45]. Oxidative stress, ubiquitination defects and mitochondrial dysfunction are commonly associated with neurodegeneration. In particular, it has been demonstrated in neural cells that in response to oxidative stress, PrP^C^ can be transformed into a PK-resistant protein, suggesting that this damage may be the initial cause of a given prion disease [46]. It has also been reported that the oxygen peroxide molecule malondialdehyde is present in the brain structures of opiate-dependent mice, and that withdrawal leads to alterations in oxidative status [47]. In addition, PrP^C^ is involved in copper transport and in cell defense mechanisms against oxidative insult, through the regulation of the intracellular CuZn superoxide dismutase activity (Cu-Zn SOD), protecting cells against heavy metals overload and subsequent oxidative stress [24,26]. Moreover, it has been suggested that overexpressed PrP^C^ could be interpreted as a molecular marker of cell Cu deficiency [48, 49,50]. Thus, if morphine withdrawal promotes an increase of ROS molecules, we can hypothesize that enhanced expression of hippocampal PrP^C^ may be considered as a phenomenon reflecting the ability of PrP^C^ to link Cu^2+^ and, in turn, to activate Cu-Zn SOD. Therefore, such a process can be considered as a hippocampal adaptive process to balance and/or to react against stress-dependent neuronal toxicity.

In our study, in hippocampal samples derived from rats during morphine withdrawal we observed the presence of two bands at approximately 60 kDa and 48 kDa, suggesting that, under particular conditions, PrP^C^ may form dimeric structures *in vivo*, which could represent homodimers arising from the PrP^C^ isoforms mono- and di-glycosylate. Current models on the role of PrP^C^ in TSEs pathogenesis also postulate the key role of PrP^C^ self-interaction in the conversion processes and in particular in the dimer formation [51]. In order to verify this hypothesis, we analyzed whether the dimeric structures of PrP^C^ were sensitive to PK. Results showed that after proteinase K digestion, the oligomeric forms were completely degraded. Although a very little portion of overexpressed PrP^C^, but not PrP^C^ in dimeric form, seems to be undegraded, it may be due to the increased PrP^C^ protein level.

Beland and coworkers [23,52] showed that homodimerization leads to a considerable increase of PrP^C^ α-cleavage and release of PrPN1 and PrPC1, with an increased PrP^C^ trafficking to the plasma membrane. They proposed that the dimerization at the plasma membrane triggers prosurvival intracellular signaling cascades and neuroprotective PrPN1 and PrPC1 formation. In our hands, rats which have been withdrew from chronic morphine showed a 16 KDa band, that could represent a PrPC1 fragment, which allows us to hypothesize the parallel formation of the soluble fragment N1.

The observed modifications of PrP^C^ induced by opiate withdrawal may have several important functional implications. Indeed, over the past fifteen years, evidence has accumulated that PrP^C^ can act as a cell surface receptor, co-receptor or ligand, able to recruit downstream signal transduction pathways [53]. In particular, antibody-mediated dimerization of PrP^C^ at the plasma membrane induces the cell survival ERK cascade and the production of ROS [54, 55]. Moreover, PrP^C^, as well as many GPI-anchored proteins, is a constitutive component of lipid rafts [3,56], specialized subcellular compartments, enriched in receptors, second messengers and effectors, which are considered “signal transduction domains”, which represent very important signaling domains on the cell surface [57].

It is now well-established that PrP^C^ can serve as a cell signalling molecule, able to mobilize transduction cascades from its location in lipid rafts in response to interactions with partners. Among the intracellular effectors of PrP^C^, authors [17,12,58] reported a role for MAP kinases ERK1/2. [59,60], Of note, Erk signalling pathway is necessary for morphine withdrawal-induced gene expression and activation of brain areas associated with the stress system [61].

Moreover, as previously demonstrated, PrP^C^ is an essential molecule for apoptotic machinery [62,63], neverthless its α-cleavage products may have a prosurvival function. The mechanisms by which morphine withdrawal may regulate PrP^C^ are not completely clarified. Recently, the responses of PrP^C^ to various stresses, including ischemia, oxidative stress, inflammation and autophagy have been reported [64].

Here we show, for the first time to our knowledge, the aggregation of PrP^C^ in a biological process not related to neurodegeneration. Our results may suggest that PrP^C^ dimerization, and its further aggregation in partially resistant oligomers, may play a role in the restoration of network homeostasis associated with withdrawal from morphine.

More studies are needed in order to explore whether the presence of PrP^C^ is necessary for the restoration of physiological state following drug withdrawal. One may further speculate whether the process is shared by other homeostatic responses, opening the fascinating perspective of a novel biological mechanism based on protein aggregation which may serve as a regulator of welfare of the brain.

## Supporting Information

S1 Fig Expression of the GPI-anchored protein Thy-1 after opiate withdrawal. Left panel. Representative immunoblots of the control GPI-anchored protein Thy-1 from hippocampi of rats after 7 and 14 days of opiate withdrawal. Samples were analyzed by Western blot, using anti-Thy-1 monoclonal antibody. Right panel. Densitometric analysis of bands from each samples, Mean ± SD.
(TIF)
