# Supplementary figures and images for: Morphine Withdrawal Modifies Prion Protein Expression in Rat Hippocampus
Source: PLoS One. 2017 Jan 12;12(1):e0169571. doi: 10.1371/journal.pone.0169571 (PMC5231345; doi:10.1371/journal.pone.0169571)

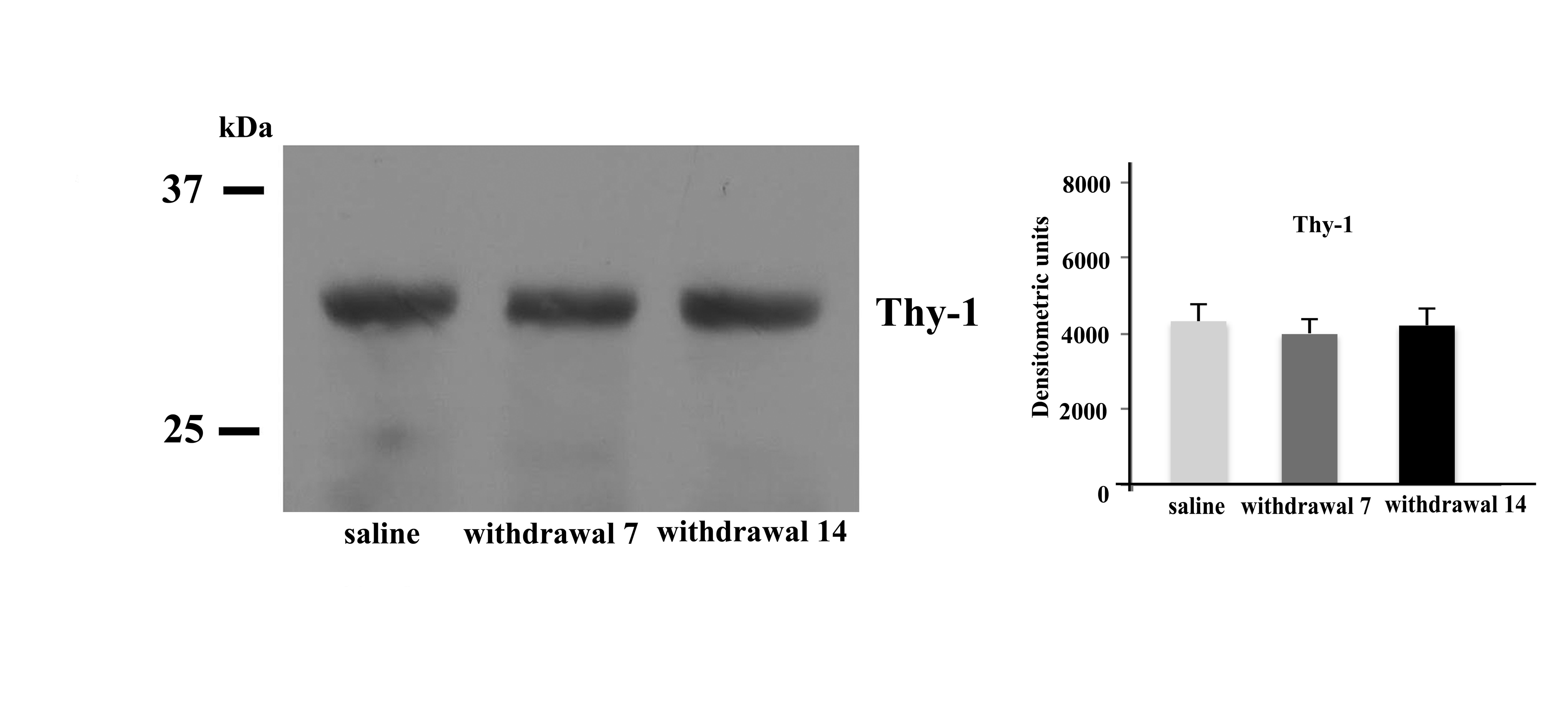

Supplement: S1 Fig — Left panel. Representative immunoblots of the control GPI-anchored protein Thy-1 from hippocampi of rats after 7 and 14 days of opiate withdrawal. Samples were analyzed by Western blot, using anti-Thy-1 monoclonal antibody. Right panel. Densitometric analysis of bands from each samples, Mean ± SD. (TIF) [file pone.0169571.s001.tif]
